# Types of Assistances of Evidence-Based Dementia Caregiving Programs: Data Results and Future Directions

**DOI:** 10.1093/geroni/igab046.026

**Published:** 2021-12-17

**Authors:** Alyssa Ciancibello, David Bass, Rachel Schaffer, Sara Powers

**Affiliations:** Benjamin Rose Institute on Aging, Cleveland, Ohio, United States

## Abstract

A key feature displayed in Best Practice Caregiving are the types of assistances. Data on 54 areas of care were collected for all 44 programs. These were analyzed through factor analysis and grouped into 19 types of assistance. Types were analyzed by the number of assistances provided, delivery method, and recipient of assistance. On average, programs delivered 11.8 types of assistances, with the most common types being Supporting CG/Individual-with-Dementia (IWD) Communication, Encouraging Positive CG-IWD Activities, and Assisting with Coping (93.2%), with the least common being Getting a Dementia Diagnosis (29.5%) and Monitoring Benefits of Services (20.5%). Assistance was delivered most often through information/referral delivery (M=11.07, SD=5.41) than direct (M=3.77, SD=4.54) or skills training (M= 7.50, SD=4.54). Results of the data show the breadth and characteristics of assistances programs provide to support caregivers of persons with dementia, along with gaps in types of assistances and future directions for programs.

